# A novel fusion partner for enhanced secretion of recombinant proteins in *Saccharomyces cerevisiae*

**DOI:** 10.1007/s00253-016-7722-2

**Published:** 2016-07-13

**Authors:** Jung-Hoon Bae, Bong Hyun Sung, Jeong-Woo Seo, Chul Ho Kim, Jung-Hoon Sohn

**Affiliations:** 1 Cell Factory Research Center, Korea Research Institute of Bioscience and Biotechnology (KRIBB), Daejeon, 34141 Republic of Korea; 2 Industrial Microbiology and Bioprocess Research Center, Korean Research Institute of Bioscience and Biotechnology, Jeongeup, 56212 Republic of Korea

**Keywords:** *Saccharomyces cerevisiae*, *VOA1*, Fusion partner, Purification

## Abstract

Expressing proteins with fusion partners improves yield and simplifies the purification process. We developed a novel fusion partner to improve the secretion of heterologous proteins that are otherwise poorly excreted in yeast. The *VOA1* (YGR106C) gene of *Saccharomyces cerevisiae* encodes a subunit of vacuolar ATPase. We found that C-terminally truncated Voa1p was highly secreted into the culture medium, even when fused with rarely secreted heterologous proteins such as human interleukin-2 (hIL-2). Deletion mapping of C-terminally truncated Voa1p, identified a hydrophilic 28-amino acid peptide (HL peptide) that was responsible for the enhanced secretion of target protein. A purification tag and a protease cleavage site were added to use HL peptide as a multi-purpose fusion partner. The utility of this system was tested via the expression and purification of various heterologous proteins. In many cases, the yield of target proteins fused with the peptide was significantly increased, and fusion proteins could be directly purified with affinity chromatography. The fusion partner was removed by in vitro processing, and intact proteins were purified by re-application of samples to affinity chromatography.

## Introduction

Owing to its generally recognized as safe (GRAS) status, the yeast *Saccharomyces cerevisiae* has been widely used as a popular workhorse for the production of various pharmaceutical and industrial proteins. The yeast expression system combines the advantages of both bacteria and higher eukaryotic cells (Kunes et al. 1987). It is a eukaryotic microorganism that can be cultured at high cell density, which facilitates large-scale fermentation. Additionally, the yeast protein secretion pathway and post-translational modification systems are similar to those of higher eukaryotic cells, although the fidelity of protein hyper-glycosylation varies according to the specific target (Buckholz and Gleeson 1991). Thus, yeast can produce various complex proteins that are native to higher eukaryotes including humans; this is a distinct advantage over production in *Escherichia coli*, as proteins lack eukaryotic modifications and are often inactive. Furthermore, secretory production of foreign proteins greatly simplifies the purification procedure and reduces the production cost compared to intracellular production.

Secretory production of heterologous protein involves the sequential processes of protein synthesis, folding and secretory trafficking. The secretion rate of overexpressed proteins is mainly dependent on the folding rate in the endoplasmic reticulum (ER) (Robinson and Wittrup 1995). Unless they fold correctly, these proteins accumulate in the ER as aggregates; this triggers the unfolded protein response and eventual degradation of proteins by an ER-associated degradation pathway (Ellgaard and Helenius 2003). Numerous approaches have been applied to enhance protein-folding rate and avoid aggregation and degradation. These include overexpression of molecular chaperones, foldases (Hackel et al. 2006),(Powers and Robinson 2007), and genes related to the secretory pathway (Carla Fama et al. 2007), (Wentz and Shusta 2007) and engineering of the leader sequence (Clements et al. 1991),(Kjaerulff and Jensen 2005).

Another way to increase protein secretion is by direct fusion of the target protein to well-secreted proteins such as human serum albumin (Kang et al. 2007), (Huang et al. 2008) protein disulfide isomerase (Kajino et al. 2000), Hsp150 protein (Sievi et al. 2003)**,** cellulose-binding domain (Ahn et al. 2004) and cell wall protein Pir4 (Andres et al. 2005). The secretion-enhancing effects of such fusions are often attributed to the improved stability and transport of target proteins.

We previously developed a translational fusion partner (TFP) technology that provides optimal fusion partners for secretory production of otherwise poorly secreted proteins in yeast (Bae et al. 2015). In the course of TFP screening from yeast genome, we discovered that the *VOA1* protein was abundantly secreted into extracellular medium when the C-terminal transmembrane domain was deleted and overexpressed under the control of the strong promoter.

In this study, we engineered C-terminally truncated Voa1p for use as a multi-functional fusion partner for secretory production of foreign proteins in yeast. Using a series of deletion variants, we found that a domain of Voa1p comprised of 28 hydrophilic amino acids (HL domain) was sufficient to enhance protein secretion. Consequently, we performed additional engineering to add a purification tag and protease cleavage site to the HL domain; this facilitated both the expression and purification of various heterologous proteins.

## Methods

### Strains and growth conditions

Haploid yeast *Saccharomyces cerevisiae* Y2805 (*Mat α pep4::HIS3 prb1 can1 his3-200 ura3-52*) (Choi et al. 1994) was used as the general host for gene expression. All yeast transformations were performed using the lithium acetate method (Gietz et al. 1995). Yeast were generally grown on YPD (1 % yeast extract, 2 % bacto peptone, and 2 % glucose). General selection of yeast transformants was performed using a UD plate media (0.67 % yeast nitrogen base without amino acids, 2 % glucose, 0.5 % casamino acids, and 2 % agar). Recombinant yeast strains were cultivated on YPDG (1 % yeast extract, 2 % bacto peptone, 1 % glucose, and 1 % galactose) to induce *GAL10* promoter in the Y2805 strain. For the fermentation of recombinant strains, a seed culture was prepared using UD broth, and transferred to a 1000-ml Erlenmeyer flask containing 200 ml of UD broth and incubated in shaking incubator for overnight at 30 °C. The cultured seed (200 ml) was inoculated into a 5-l jar fermenter (Kobiotech, Seoul, Korea) containing 1800 ml of fermentation medium (4 % yeast extract, 1 % bacto peptone, and 2 % of glucose). When glucose was completely exhausted, a feeding medium containing 300 g of glucose, 300 g of galactose, and 150 g of yeast extract (per liter) was added. The hourly feeding rate was manually increased from 2 to 10 g/l of carbon source according to cell growth. Ammonia solution was used to maintain the fermentation at pH 5.5. *E. coli* DH5α [*F* ^*−*^ *lacZΔM15 hsdR17(r- m-) gyrA36*] was used for general recombinant DNA procedures.

### Vector construction

The primers used in this study are summarized in Table 1. To construct the plasmid YGaT41, which contains an intact *VOA1* gene under the control of the *GAL10* promoter, the open reading frame (ORF) of *VOA1* was amplified from *S. cerevisiae* Y2805 genomic DNA with polymerase chain reaction (PCR) primers, a sense primer (T4F) containing a *Bam*HI site, and an antisense primer (T41R) containing a *Sal*I site. The amplified *VOA1* ORF was digested with *Bam*HI-*Sal*I and sub-cloned into the *Bam*HI-*Sal*I sites of YEGα-HIR525 (Choi et al. 1994). To construct incremental C-terminally truncated *VOA1* gene expression vectors, four antisense primers (T42R–T45R) were designed and used to amplify *VOA1* variants in combination with the T4F primer. The amplified partial *VOA1* gene fragments were cloned into the *Bam*HI-*Sal*I site of YGaT41 and the resulting plasmids were named YGaT42, 43, 44, and 45.

**Table 1:** Summary of primers used for plasmid construction

Primer	Sequence
T4F	ATCGGATCCATGGTGTTCGGTCAGCTG
GAL100	GTATATGGTGGTAATGCCATG
GT50R	GTCATTATTAAATATATATATATATATATTGTCACTCCGTTCAAGTCGAC
IL2F	CTCGCCTTAGATAAAAGAGCACCTACTTCAAGTTCTAC
IL2R	GTCACTCCGTTCAAGTCGACCTAAGTTAGTGTTGAGATG
LNK-R	CTTTTATCTAAGGCGAGGCCAGCAGAGGCCGAGGCGGCCACCCCTTCTTCTTTA
HDK-R	GTCATCGTCACCGTGGTGATGGTGATGATGGCTCAAAGTCTCTT
HL-F	GTTATTAACTCTCTTGGTTG
HL-GT50R	CACTCCGTTCAAGTCGACTTAGTGGTGATGGTGATGATGG
H119	TCTTTTATCTAAGGCGAGAAAAGCCCAACCAAGAG
H120	TCTTTTATCTAAGGCGAGCTCTTCTGTTGCATATTC
H121	TCTTTTATCTAAGGCGAGATCATCGTCGCCTTCTTTAC
T41R	ATCGGTCGACTTAATTGTTTTTTTTTATTGG
T42R	ATCGGTCGACTTAATCATCGTCGCCTTCTTTAC
T43R	ATCGGTCGACTTACTCTTCTGTTGCATATTC
T44R	ATCGGTCGACTTAAAAAGCCCAACCAAGAGAG
T45R	ATCGGTCGACTTAGTCGCCAGATTTATCTTCC
H165	ATCGGTCGACTTAGTCGCCAGATTTATCTTCC
H310	GGTGACGATGACGATAAGTCTGTGAGTGAAATACAGC
H311	CACTCCGTTCAAGTCGACTTACTGGGATTTAGCTTTAG
H410	GGTGACGATGACGATAAGAACTCCGACTCCGAGTGTC
H616	CTCGCCTTAGATAAAAGAAACTCCGACTCCGAGTGTC
H617	ACCAAGAGAGTTAATAACTCATCTCAGCTCCCACCAC
H618	CATCGTCACCGTGGTGATGGTGATGATGGCTCAAAGTCTCTTCTG
H619	TCACCACGGTGACGATGACGATAAGAACTCCGACTCCGAGTGTC
H620	GTCACCATCTTCATCTTCTCTTTTATCTAAGGCGAGG
H621	CCTCGCCTTAGATAAAAGAGAAGATGAAGATGGTGAC

To fuse the human interleukin-2 (hIL2) gene to the truncated *VOA1* construct in YGaT42, the partial *VOA1* gene was amplified with a sense primer (GAL100) recognizing the *GAL10* promoter and an antisense primer (H121) that recognizes sequences in the YGaT42 vector. The hIL2 gene was amplified with sense primer IL2F, which recognizes sequences in the *VOA1* gene that are complementary to those of the H121 primer, and an antisense primer (IL2R) that contains part of the *GAL7* terminator sequence. The amplified PCR fragments were annealed to a single fragment by overlap-extension PCR using GAL100 and GT50R primers. GT50R primer runs in the antisense direction and contains 50 nucleotides of *GAL7* terminator sequence. The resulting PCR product was flanked with 100 bp of *GAL10* promoter sequence and 50 bp of *GAL7* terminator sequence. The recombinant *S. cerevisiae* Y2805 strain was directly constructed by co-transformation with the fused PCR product and *Bam*HI-*Sal*I-digested YGaT41 vector backbone. Circular topology of plasmid is restored in host cells by homologous recombination of a linearized vector with a DNA fragment that contains sequences that overlapping sequences at each end (Kunes et al. 1987). The resulting plasmid was named YGaT42-IL2. To construct an *S. cerevisiae* Y2805 strain transformed with YGaT43-IL2 and YGaT44-IL2 vectors, H120 and H119 primers were used instead of H121 primer. The YEGα-EGF plasmid was constructed by subcloning a chemically synthesized *hEGF* gene (sequence derived from a public database, www.ncbi.nlm.nih.gov/genbank) into the *Xba*I and *Sal*I sites of the YEGα-HIR525 plasmid. To construct YEGα-HL50-EGF plasmid, the MFα pre-pro peptide gene, the HL peptide gene, and *hEGF* were amplified from YEGα-HIR525, YGaT41, and YEGα-EGF with GAL100/LNK-R, H221/HDK-R, and H410/GT50R primer sets, respectively. These fragments contain 17 or 18 overlapping nucleotides in order to facilitate their contiguous assembly. Using overlap-extension PCR with the GAL100/GT50R primer set, the order of the resulting single fragment was: MFα pre-pro peptide-HL peptide-hEGF. To construct the YEGα-EGF-HL plasmid, the HL peptide gene was amplified with the HL-F/HL-GT50R primer set, and *hEGF* was amplified with the H616/H617 primer set. These fragments were fused with the MFα pre-pro peptide coding sequence that was amplified with the GAL100/LNK-R primer set. Using overlap-extension PCR with the GAL100/GT50R primer set, the order of the single fragment was MFα pre-pro peptide-hEGF-HL. Recombinant *S. cerevisiae* Y2805 strains transformed with YEGα-HL50-EGF or YEGα-EGF-HL50 were directly constructed by co-transformation with the fused PCR products and *EcoR*I/*Sal*I-digested YEGα-EGF vector backbone. YEGα-EGF, YEGα-HL50-EGF, and YEGα-EGF-HL50 vectors contain linker peptide (AASASAGLALD**KR**) following the MFα pre-pro peptide. To construct the YEGα-HL37-EGF plasmid, the MFα-HL peptide coding sequence was amplified with the GAL100/H618 primer set, and the hEGF gene was amplified from YEGα-HL50-EGF plasmid with the H619/GT50R primer set. These fragments were fused with overlap-extension PCR using the GAL100/GT50R primer set and then cloned into the *Eco*RI/*Sal*I sites of YEGα-HL50-EGF. The MFα pre-pro peptide coding sequence amplified with the GAL100/H620 primer set and the HL-hEGF gene amplified with H621/GT50R primer set from YEGα-HL37-EGF plasmid, were fused using overlap-extension PCR with the GAL100/GT50R primer set. This product was then cloned into the *Eco*RI/*Sal*I sites of YEGα-HL50-EGF to make YEGα-HL28-EGF. YEGα-HL28-EXD4 and YEGα-HL28-IGF plasmids were constructed by replacing the hEGF gene of YEGα-HL28-EGF plasmid with each gene synthesized at Bioneer (Daejeon, Korea) by in vivo recombination.

### Protein analysis and purification

To analyze the secreted proteins in test tube culture, recombinant cells containing a foreign protein expression vector were cultivated in 3 ml of YPDG broth media for 40 h at 30 °C and then 0.6 ml of culture supernatant was mixed with 0.4 ml of cold acetone. After a 2-h incubation at −20 °C, proteins were precipitated by centrifugation for 15 min at 10,000×*g*. The pellet was freeze-dried and resuspended in 1× SDS-PAGE sample buffer (Bio-Rad, Hercules, CA, USA) and run on 10–12 % of Tris-glycine or Tricine gels under denaturing conditions. To analyze the secreted proteins from fed-batch fermentation, 5–10 μl of culture supernatant was directly used for SDS-PAGE after mixing with 2× SDS-PAGE sample buffer, and then stained with Coomassie blue. Total intracellular yeast protein was prepared from the cells by post-alkaline extraction (Bae et al. 2015). A polyclonal antibody to hIL-2 (R&D Systems Inc., Minneapolis, MN, USA) and an anti-goat IgG alkaline phosphatase conjugate (Sigma Chemical Co., St. Louis, MO, USA) was used for western blot analysis. hEGF protein fused to the HL-peptide was purified by metal affinity chromatography on a nickel-NTA agarose column (Promega, Wisconsin, USA) using medium pressure liquid chromatography (Bio-Rad). Fermentation broth was filtered with a 0.1-μm Sartoclear filter (Sartorius, Goettingen, Germany), and concentrated by ultrafiltration with 10 K NMWC Quick-stand (Amersham-Pharmacia Biotech, Piscataway, NJ USA) using buffer A [50 mM Tris-HCl (pH 8.0), 0.5 M NaCl]. The concentrated solution was loaded onto the column at a flow rate of 1 ml/min and proteins were eluted in a gradient of buffer B [50 mM Tris-HCl (pH 8.0), 0.5 M NaCl, 0.5 M imidazole]. The fractions containing proteins of interest were concentrated, and buffer B was replaced with EK buffer [20 mM Tris-HCl (pH 8.0), 50 mM NaCl, and 2 mM CaCl2] by ultrafiltration with 10 K MWCO Amicon Ultra centrifugal filter device (Millipore, Massachusetts, USA). To remove the HL peptide from the fused protein, enterokinase (Invitrogen, USA) was added to the protein solution (one unit per mg of fused protein) and incubated at 16 °C for 15 h. The intact hEGF protein was obtained by repeating metal affinity chromatography on the nickel-NTA agarose column using medium pressure liquid chromatography. The molecular weight of the purified hEGF protein was determined by using a 4700 Proteomic Analyzer (Applied Biosystems, Framingham, MA, USA) at Korea Basic Science Institute (Daejeon, Korea). The bioactivity of hEGF was determined with a lymphocyte proliferation assay using the EL-4 mouse T-lymphocyte cell line and a Cell Proliferation ELISA, BrdU kit (Roche, Germany).

## Results

### Determination of the optimal *VOA1* fragment for extracellular secretion

**Fig. 1:**
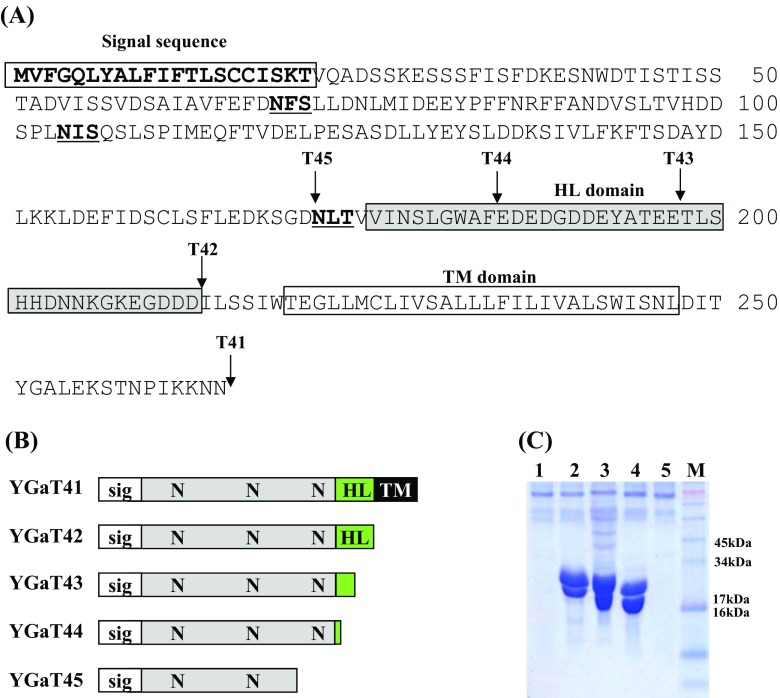
Expression analysis of C-terminally truncated *VOA1* derivatives. **a** The predicted amino sequence and domains encoded by the *VOA1* gene. Truncation sites are indicated by *arrows*. Glycosylation sites are highlighted in *bold*. **b** Schematic diagram of serially deleted *VOA1* genes. *Sig* signal peptide, *N* N-linked glycosylation site, *HL* hydrophilic domain, *TM*: transmembrane domain. **c** Expression levels of *VOA1* derivatives were analyzed by SDS-PAGE. *Lanes 1–5* culture broth of *S. cerevisiae* transformed with YGaT41–YGaT45 vector, respectively; *lane M* pre-stained protein size marker (Invitrogen)

Voa1p is an ER membrane protein that is postulated to be one of five V0 assembly factors for vacuolar ATPase (Ryan et al. 2008). Voa1p consists of 265 amino acids containing a secretion signal peptide, three glycosylation sites, a proposed hydrophilic domain (HL), and a transmembrane domain (TM) (Fig. 1a). In normal conditions, this protein is retained in the ER membrane but a C-terminally truncated partial Voa1p (213 amino acids) was secreted into the culture medium at high levels when expressed under control of the *GAL10* promoter.

To determine the optimal domains required for secretion of Voa1p, we expressed five incremental C-terminal deletion mutants in yeast under the control of a *GAL10* promoter. As shown in Fig. 1, no proteins were detected in the culture supernatant of the YGaT41 strain expressing full-length Voap1 (Fig. 1c, lane 1). In contrast, a high amount of truncated *VOA1* protein was secreted from cells expressing YGaT42, YGaT43, and YGaT44 (Fig. 1c, lanes 2–4). The Voa1p derivatives were visible as two bands due to glycosylation events. This was confirmed by the disappearance of the higher molecular weight band and concomitant increased intensity of the low molecular weight band following treatment with the Endo-H glycosylase (data not shown). Interestingly, further truncation of the Voa1p sequence in YGaT45 (which encodes a protein with no HL domain and removes a glycosylation site), did abrogated secretion of the protein. From these data, we conclude that hypersecretion of Voa1p, is facilitated by removal of the TM domain, and is to some extent dependent on the structural portion of Voa1p that contains all three glycosylation sites.

### Utilization of Voa1p derivatives as a fusion partner

**Fig. 2:**
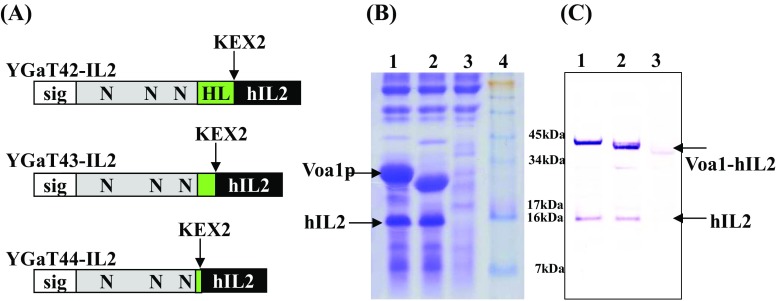
Expression analysis of *VOA1* derivatives fused with *hIL2*. **a** Schematic representation of *VOA1*-*hIL2* fusion proteins. *Sig* signal peptide, *N* N-linked glycosylation site, *HL* hydrophilic domain. **b** Confirmation of hIL2 expression by SDS-PAGE analysis. *Lanes 1–3* culture broth of *S. cerevisiae* transformed with YGaT42-IL2, YGaT43-IL2, and YGaT44-IL2 vector, respectively; *lane M* pre-stained protein size marker (Invitrogen). **c** Western blot analysis of intracellular proteins. *Lanes 1–3* cell extract of recombinant strains carrying YGaT42-IL2, YGaT43-IL2, and YGaT44-IL2 vector, respectively

To test Voa1p derivatives as fusion partners that could improve the secretion of otherwise poorly secreted heterologous proteins from yeast, we selected human interleukin-2 (hIL2). First, the hIL2 gene was fused to the ends of the Voa1p derivatives encoded in the YGaT42, YGaT43, and YGaT44 vectors. A yeast dipeptidyl protease Kex2p recognition sequence (Leu-Asp-Lys-Arg) was added between Voa1p derivatives and hIL2 to facilitate in vivo processing (Mizuno et al. 1988). Schematic diagrams of each fusion gene construct are shown in Fig. 2a. After cultivation of each recombinant strain, concentrated culture supernatants and intracellular fractions were analyzed using SDS-PAGE and Western blotting, respectively (Fig. 2b, c). Two bands corresponding to C-terminally truncated Voa1p and correctly processed hIL2 were identified in the cases of YGaT42-IL2 and YGaT43-IL2, respectively (Fig. 2b, lanes 1 and 2). Both vectors contained at least some hydrophilic residues of the Voa1p HL domain. In contrast, no Voa1p or hIL2 bands were observed following induction of YGaT44-IL2, which encodes a variant that completely lacks hydrophilic residues of the HL domain (Fig. 2b, lane 3). In intracellular fraction, unprocessed Voa1p-hIL2 fusion protein bands were detected in all cases but processed hIL2 bands were detected only in the cases of YGaT42-IL2 and YGaT43-IL2 (Fig. 2c, lanes 1 and 2). Therefore, fusion protein from YGaT44-IL2 may not reach the Golgi complex and would be gradually degraded by the ER quality control system. The HL domain was not crucial for the secretion of C-terminally truncated Voa1p from YGaT44 (Fig. 1c, lane 4). However, in the context of the C-terminally truncated Voa1p and hIL2 fusion protein, hydrophilic residues of the HL domain was critical for secretion. As shown in Fig. 2b, c, hIL2 proteins was hardly detected in extra and intracellular fraction without the HL domain. Thus, we conclude that the HL domain improves the solubility of fusion proteins in the ER, and thus facilitates trafficking between ER and Golgi complex.

### Utilization of HL domain as a fusion peptide to improve the solubility of target proteins

**Fig. 3:**
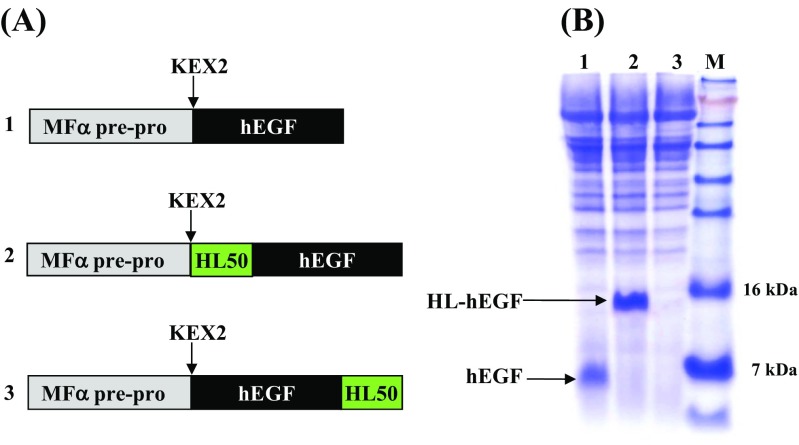
Effects of HL50 tagging on the expression of hEGF. **a** Schematic representation of the hEGF secretion cassette. **b** SDS-PAGE analysis of culture broth of *S. cerevisiae* transformed with YEGα-EGF (*lane 1*), YEGα-HL50-EGF (*lane 2*), and YEGα-EGF-HL50 (*lane 3*), respectively; *lane M* pre-stained protein size marker (Invitrogen)

Based on the above-mentioned results, we hypothesized that acidic amino acids within the HL domain might contribute to the increased secretion of proteins, since the solubility of a protein is closely related to its net charge (Zhang et al. 2004) (Trevino et al. 2007). For general application, a metal affinity purification tag and an EK recognition sequence were added to the end of HL peptide resulting HL50. To further investigate the positional effects of the HL domain, we fused HL50 to either the N-terminus or C-terminus of human epidermal growth factor (hEGF) (YEGα-HL50-EGF and YEGα-EGF-HL50), respectively (Fig. 3a). To focus on the effects of HL domain, secretion of HL50-tagged hEGF was controlled by a generally used yeast secretion signal (the mating factor α pre-pro peptide) instead of Voa1p signal. As shown in Fig. 3b, N-terminal tagging of hEGF with HL50 improved the secretion of fusion proteins (Fig. 3b, lane 2) ~10-fold compared to untagged proteins (Fig. 3b, lane 1). On the other hand, hEGF tagged with HL50 at the C-terminus was poorly secreted (Fig. 3b, lane 3). Use of the N-terminal HL peptide tag is also desirable as it facilitates the removal of tags from fusion proteins. This is since commonly used tags are cleaved by sequence-specific endoproteases such as EK, TEV, and Factor Xa that cleave after their recognition sites; thus, N-terminal tags are removed without leaving residual peptides on the target proteins.

### Engineering of HL peptide as a general fusion tag

**Fig. 4:**
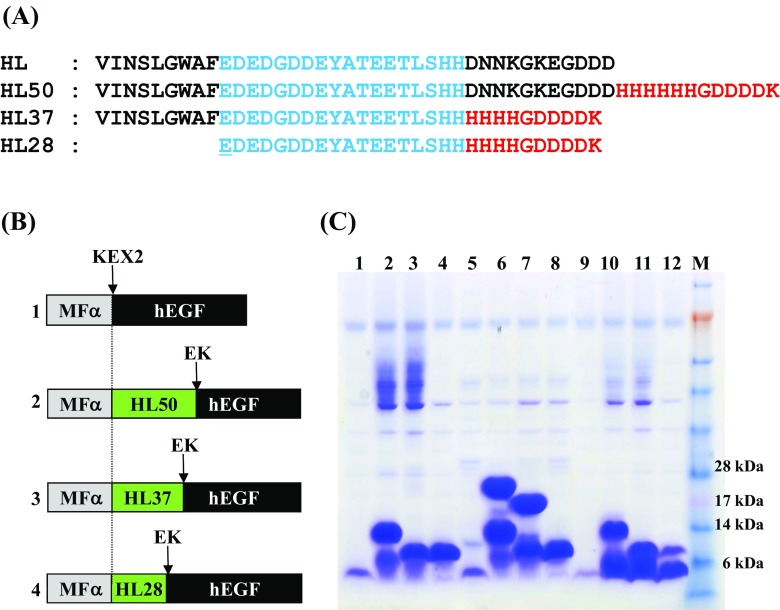
Expression of hEGF using modified HL peptides. **a** Amino acid sequence of modified HL peptides. **b** Schematic representation of the hEGF secretion cassette. **c** SDS-PAGE analysis of culture broth of *S. cerevisiae* transformed with YEGα-EGF (*lanes 1, 5, 9*); YEGα-HL50-EGF (*lanes 2, 6, 10*); YEGα-HL37-EGF (*lanes 3, 7, 11*); and YEGα-HL28-EGF (*lanes 4, 8, 12*); respectively. *Lanes 5–8* after deglycosylation, *lanes 9–12* after digestion with EK, *lane M* pre-stained protein size marker (Invitrogen)

To optimize the HL peptide as a general fusion tag, three HL variant peptides, HL50, HL37, and HL28, were tested for their ability to promote hEGF secretion. As shown in Fig. 4a, the HL50 construct contains a metal affinity purification tag and EK recognition sequence (HHHHHHGDDDDK) at the end of the HL peptide. For HL37, 11 amino acids of the HL peptide (DNNKGKEGDDD) were replaced with 10 amino acids (HHHHGDDDDK). To minimize the size of HL domain without altering its net charge, nine nonpolar amino acids (VINSLGWAF) located at the N-terminus of HL37 were removed in order to yield construct HL28. According to the positional preference (Fig. 3), the three HL variants were fused to the N-terminus of hEGF, and the MF α pre-pro peptide was added to promote secretion of all fused proteins. The secretion-enhancing effect of the HL variant peptide was still maintained after modification (Fig. 4c, lane 3) and truncation (Fig. 4c, lane 4). Because there are no possible glycosylation sites in the HL50 peptide or hEGF, we infer that the high molecular weight smear bands formed by YEGα-HL50-EGF and YEGα-HL37-EGF (Fig. 4c, lanes 2, 3) were due to hyper-glycosylation of incompletely processed MF α pro peptide. To confirm the presence of hyper-glycosylation, secreted fusion proteins were re-analyzed after treatment with Endo-H (Fig. 4c, lanes 5–8). As expected, unprocessed fusion bands appeared after deglycosylation in YEGα-HL50-EGF and YEGα-HL37-EGF (Fig. 4c, lanes 6, 7). However, neither incomplete processing by Kex2p nor hyper-glycosylation were found in the case of YEGα-HL28-EGF, suggesting that removal of nine nonpolar amino acids (VINSLGWAF) facilitated complete processing of HL28-hEGF by Kex2p (Fig. 4c, lane 8). To test the susceptibility of each HL peptide to in vitro endoprotease digestion, secreted fusion proteins were treated with EK. In contrast to the HL50-hEGF and HL37-hEGF fusion proteins, which were poorly digested (Fig. 4c, lanes 10 and 11), about 80 % of HL28-hEGF was digested, yielding intact hEGF (Fig. 4c, lane 12). These results clearly demonstrate that HL28 is a good candidate as a general fusion peptide for secretory expression of heterologous proteins.

### Characterization of yeast recombinant hEGF

**Fig. 5:**
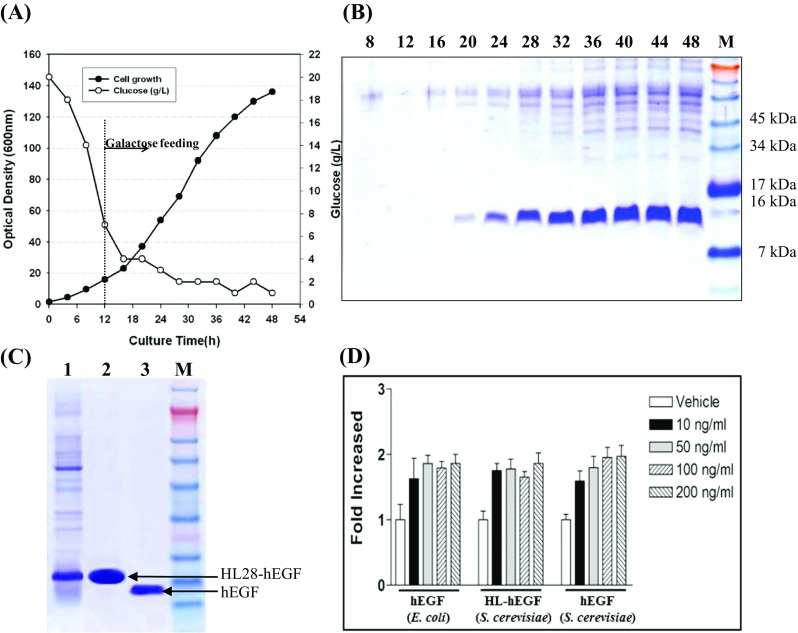
Confirmation of recombinant hEGF expression. **a** Time profiles of fed-batch fermentation of *S. cerevisiae* expressing YEGα-HL28-EGF. **b** SDS-PAGE analysis of culture supernatants. Samples of culture supernatants (10 μl) at the indicated times were analyzed. **c** SDS-PAGE of the purified hEGF. *Lane 1* after ultrafiltration of fermentation broth, *lane 2* after Ni-NTA affinity chromatography, and *lane 3* purified hEGF after EK digestion. **d** Bioactivity assay of the purified hEGF. The EL-4 cell line was cultured in the presence of the indicated amounts of hEGF, and cell proliferation was analyzed following bromodeoxyuridine (BrdU) labeling

A recombinant Y2805 strain harboring YEGα-HL28-EGF was cultured in a 5-l jar fermenter under fed-batch fermentation conditions. Samples were taken over a 48-h culture period and supernatants were analyzed by SDS-PAGE (Fig. 5a, b). Using hEGF produced by *E. coli* as a standard, the amount of secreted HL28-hEGF fusion protein was ~400 mg/l. The HL28-hEGF fusion protein was first directly purified by Ni-NTA affinity chromatography (Fig. 5c, lane 2). Following removal of HL28 using EK digestion, intact hEGF was purified by Ni-NTA affinity chromatography (Fig. 5c lane 3). N-terminal amino acid sequencing and mass analysis confirmed that the purified hEGF was intact (data not shown). The biological activities of purified intact hEGF and HL28-fused hEGF were characterized using an in vitro proliferation assay. Both constructs had growth-stimulating activity comparable to that of commercial hEGF produced in *E. coli* (Fig. 5d). This suggests that the biological activity of hEGF is not perturbed by N-terminal fusion of HL28.

### Application of HL28 to enhance protein secretion

**Fig. 6:**
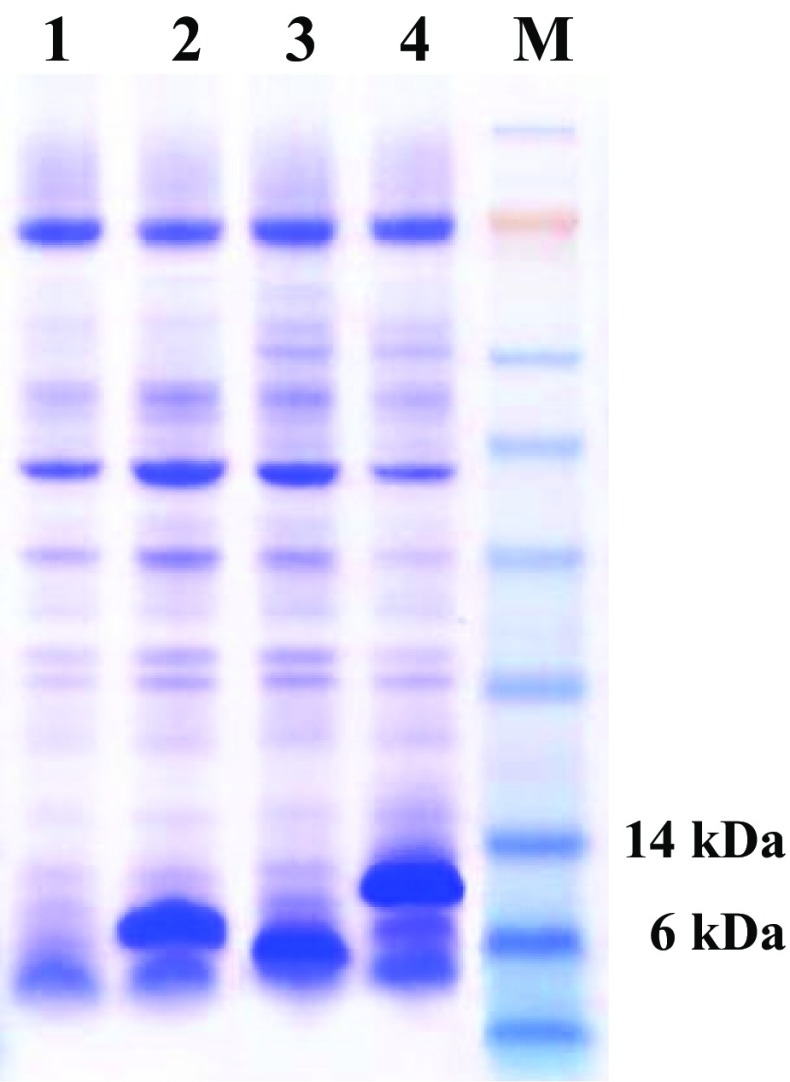
Expression of EXD-4 and hIGF-1 using HL28. SDS-PAGE analysis of culture broth of *S. cerevisiae* transformed with YEGα-EXD4 (*lane 1*), YEGα-HL28-EXD4 (*lane 2*), YEGα-IGF (*lane 3*), and YEGα-HL28-IGF (*lane 4*), *lane M* pre-stained protein size marker (Invitrogen)

We next selected two poorly secreted, but pharmaceutically important proteins to test the ability of the HL28 peptide to enhance secretion. Exendin-4 (EXD-4), a glucagon-like peptide-1 receptor agonist used for the treatment of type 2 diabetes, and human insulin-like growth factor type 1 (hIGF-1), which is used to treat type 1 and 2 diabetes were expressed under fusion with or without HL28 peptide. Secretion of recombinant proteins was ensured by expression of the MF α pre-pro peptide under the control of *GAL10* promoter (Fig. 6). EXD-4 was not detected in the culture supernatant when expressed without HL28 (Fig. 6, lane 1) but an obvious protein band corresponding to the expected size (7.4 kDa) of the fusion protein was formed after tagging the N-terminus of EXD-4 with HL (Fig. 6, lane 2). Similarly, HL28 fusion robustly increased secretion of hIGF-1 compared to untagged hIGF-1 (Fig. 6, lanes 3 and 4). These data strongly suggest that the HL28 peptide will find utility in the production of poorly secreted recombinant proteins in yeast, and will also enhance production and simplify the process of protein purification.

## Discussion

A generally preferred method for enhanced secretion and efficient purification of recombinant proteins is to express them with fusion partners. There have been numerous reports on the development and application of fusion tags (Kang et al. 2007), (Huang et al. 2008),(Kajino et al. 2000), (Sievi et al. 2003)**,** (Ahn et al. 2004), (Andres et al. 2005). In this regard, we developed a novel fusion partner by using the *S. cerevisiae* ER membrane protein, Voa1p. In a previous report (Bae et al. 2015), we discovered that Voa1p is secreted at high levels (grams per liter) into the extracellular medium by *S. cerevisiae* when the TM domain was removed. Therefore, we expected that the truncated protein would have great potential as a fusion partner for soluble expression of target proteins in *S. cerevisiae.* Although high molecular weight fusion partners such as human serum albumin are widely used, low molecular weight fusion partners are usually preferred for economic reasons, since the smaller fusion partner requires less cellular resources and reduces the burden for the host cell. Here, we identified a hydrophilic peptide (HL peptide) consisting of 28 amino acid that is responsible for the improvement of target protein secretion. The original HL peptide contained high portion of charged amino acids (14 acidic amino acids and 4 basic amino acids) and modified HL peptide contained more charged amino acids (13 acidic amino acids and 7 basic amino acids) owing to six histidine and EK cleavage sites. Since the HL28 peptide is not large enough to assume significant tertiary structure, we infer that its ability to enhance secretion of fusion proteins is due to the high portion of charged amino acids, rather than the overall conformation of the peptide.

A large net negative charge increases protein solubility by increasing electrostatic repulsion forces between nascent proteins (Zhang et al. 2004) (Trevino et al. 2007). Consistent with this, the net negative charges of hEGF (−3.9 at pH 7.0), EXD-4 (−2.9), and hIGF-1 (0.7) increased to −14.9, −13.8, and −10.25 by addition of HL28, respectively. Therefore, modification of passenger protein net negative charge may increase secretion efficiency by affecting to protein solubility in the ER, or by altering trafficking to Golgi.

In addition to the proteins reported in the “Results” section, HL peptide was further applied to the expression of other clinically relevant proteins, such as human parathyroid hormone (hPTH) and immunoglobulin (Ig). Fusion of HL28 to the N-terminus of hPTH increased expression about fivefold, but did not alter the amount of Ig secretion (data not shown). We also applied the HL28 peptide for the expression of highly expressed industrial proteins, such as lipase B from *Candida antarctica* (*CalB*) and cellobiohydrolase 1 (*CBH1*) from *Trichorderma reesei* in order to further increase the expression of these proteins. As in the case of Ig, the expression of these proteins was not affected by HL28 tagging. Considering the likely mechanism of action for the HL28 peptide, these results were expected. Because HL28 peptide increases the secretion of passenger proteins by increasing the net negative charge of fused proteins, the net charge of large proteins such as Ig, *CalB,* and *CBH1* is hardly affected by HL28-tagging when compared to small proteins such as hEGF, hIGF-1, and EXD-4.

Although both N-terminal and C-terminal fusion partners can be used with heterologous proteins, N-terminal tagging is more favorable for downstream processes such as endoprotease treatment and protein purification. In most of the tested cases, the secretion-enhancing effect of the HL28 peptide was clearly dependent on the tagging position. Although N- and C-terminal HL tags increased the net negative charge of fusion proteins to a similar degree, N-terminal fusions were more robustly secreted. Therefore, we believe that, in addition to altering net charge, there are other properties of HL28 that affect folding and trafficking of passenger proteins.

In summary, we have developed a novel fusion partner that enhances the secretion of fused proteins and simplifies the purification process. This system will be useful for secretory production of heterologous proteins (particularly small peptide proteins) in *S. cerevisiae.*
